# Non-oncogenic roles of TAp73: from multiciliogenesis to metabolism

**DOI:** 10.1038/cdd.2017.178

**Published:** 2017-10-27

**Authors:** Alice Nemajerova, Ivano Amelio, Jakob Gebel, Volker Dötsch, Gerry Melino, Ute M Moll

**Affiliations:** 1 Department of Pathology, Stony Brook University, Stony Brook, NY 11794, USA; 2 Medical Research Council, Toxicology Unit, Leicester University, Hodgkin Building, Lancaster Road, PO Box 138, Leicester LE1 9HN, UK; 3 Institute of Biophysical Chemistry and Center for Biomolecular Magnetic Resonance, Goethe University, Frankfurt, Germany; 4 Department of Experimental Medicine and Surgery, University of Rome ‘Tor Vergata’, Rome 00133, Italy

## Abstract

The p53 family of transcription factors (p53, p63 and p73) covers a wide range of functions critical for development, homeostasis and health of mammals across their lifespan. Beside the well-established tumor suppressor role, recent evidence has highlighted novel non-oncogenic functions exerted by p73. In particular, p73 is required for multiciliated cell (MCC) differentiation; MCCs have critical roles in brain and airways to move fluids across epithelial surfaces and to transport germ cells in the reproductive tract. This novel function of p73 provides a unifying cellular mechanism for the disparate inflammatory and immunological phenotypes of p73-deficient mice. Indeed, mice with *Trp73* deficiency suffer from hydrocephalus, sterility and chronic respiratory tract infections due to profound defects in ciliogenesis and complete loss of mucociliary clearance since MCCs are essential for cleaning airways from inhaled pollutants, pathogens and allergens. Cross-species genomic analyses and functional rescue experiments identify TAp73 as the master transcriptional integrator of ciliogenesis, upstream of previously known central nodes. In addition, TAp73 shows a significant ability to regulate cellular metabolism and energy production through direct transcriptional regulation of several metabolic enzymes, such as glutaminase-2 and glucose-6 phosphate dehydrogenase. This recently uncovered role of TAp73 in the regulation of cellular metabolism strongly affects oxidative balance, thus potentially influencing all the biological aspects associated with p73 function, including development, homeostasis and cancer. Although through different mechanisms, p63 isoforms also contribute to regulation of cellular metabolism, thus indicating a common route used by all family members to control cell fate. At the structural level, the complexity of p73's function is further enhanced by its ability to form heterotetramers with some p63 isoforms, thus indicating the existence of an intrafamily crosstalk that determines the global outcome of p53 family function. In this review, we have tried to summarize all the recent evidence that have emerged on the novel non-oncogenic roles of p73, in an attempt to provide a unified view of the complex function of this gene within its family.


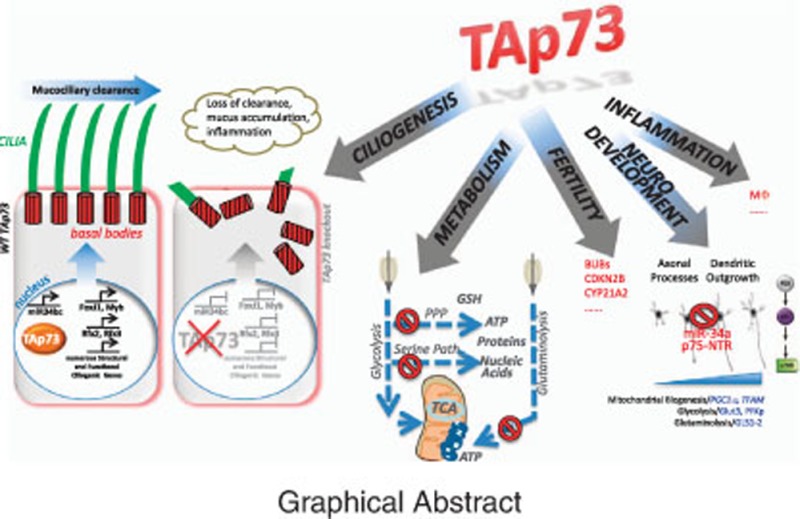


## Facts

1. Chronic respiratory tract infections of p73-deficient mice are due to loss of mucociliary clearance as the result of severe ciliary defects.
2. TAp73 is essential for basal body docking and cilium formation.
3. TAp73 transcriptionally controls a host of key regulators and structure/function genes critical for multiciliogenesis, upstream of FoxJ1, Rfx2, Rfx3 and miR34bc.
4. TAp73 controls key aspects of cellular metabolism to help coping with stress.
5. Ability to promote antioxidant defense is a central aspect for cancer- and development-related functions of TAp73.
6. p73 can form heterotetramers with p63 that are more stable than the individual homotetramers.
7. In healthy oocytes TAp63*α* forms closed, transcriptionally inhibited dimers. DNA damage-mediated phosphorylation rapidly activates its killing activity by enabling the formation of active tetramers.

## Open questions

1. Is p73 involved in chronic obstructive pulmonary disease, and how?
2. Is p73 function in airway multiciliogenesis associated with its tumor suppressor role in lung cancer?
3. Does TAp73-dependent metabolic regulation participate in the multiciliogenesis process?
4. Is there a specific function for p73/p63 heterotetramers?

P73 belongs to the p53 family of transcription factors.^1^ The *TP73* locus encodes two classes of isoforms, each undergoing further C-terminal exon splicing. The P1 promoter yields TAp73 isoforms containing the N-terminal *t*rans*a*ctivation (TA) domain.^2^ The P2 promoter yields N terminally truncated isoforms (ΔNp73) that act as dominant-negative inhibitors of p53/TAp63/TAp73. Both groups of isoforms can undergo alternative splicings at the 3′ of the mRNA giving rising up to seven different C-terminal informs, named *α*, *β*, *γ*, *δ*, *ε*, *ζ* and *η*,^3, 4^ which can be coexpressed or differently represented in a tissue-specific manner.^5^ Although weaker than p53, TAp73 is a tumor suppressor.^6, 7, 8^ Notably, aged TAp73-specific KO mice (‘TAp73KO’, missing exons 2 and 3) develop lymphoma and lung cancer,^9^ and in humans small-cell lung cancer is characterized by recurrent rearrangements in TP73 leading to oncogenic activation.^10^ However, p73’s primary role is in development and homeostasis of specific tissues. Global *Trp*73KO mice (‘p73KO’, replacing exons 5 and 6 with a neomycin cassette), missing both isoform classes, exhibit a mysterious and seemingly diverse phenotype that had been lacking a clear cellular and molecular basis for many years.

In this review, we will discuss the recent evidence regarding TAp73's contribution to cellular and organ development and homeostasis. In particular, we will describe the newly discovered role of TAp73 as the master regulator of motile multiciliogenesis, with its associated implications for the complex phenotype displayed by p73-deficient animal models. We will then analyze the role of TAp73 in the regulation of cellular metabolism in cancer and normal tissues in an attempt to provide a unified view of the homeostatic roles of TAp73. Finally, we will cover the structural aspects of TAp73 heterotetramerization properties, which carry important implications for TAp73 functions in different tissues and conditions.

## TAp73 deficiency causes profound ciliogenesis defects in airways

p73KO mice have severe hydrocephalus with cortical hypoplasia and hippocampal dysgenesis, and sensory vomeronasal abnormalities.^2^ One underlying cause for the grave brain phenotype is the severely impaired self-renewal of neural stem/progenitor cells during embryonic and adult neurogenesis.^11, 12, 13, 14^ However, this fails to *fully* explain the severe hydrocephalus that these mice have. Moreover, p73 ablation also leads to runting, airway infections and female and male fertility problems. Recently, four labs including ours identified the unifying mechanism that explains these multiorgan defects.^15, 16, 17, 18^ While searching for a common underlying theme to explain these disparate p73KO phenotypes, we noticed a strong resemblance to human primary ciliary dyskinesia (PCD) and mouse models of ciliopathies. This allowed us to identify that p73 has a key role in motile multiciliogenesis.

Cilia are highly conserved microtubule-based surface organelles with essential functions in animal cells. Most vertebrate cells can form a single, *im*motile primary cilium that senses mechanical or chemical stimuli via Hedgehog signaling. However, distinct epithelia – those lining airways, brain ependyma, the female oviduct and the testicular efferent duct – undergo multiciliogenesis by amplifying their centrioles to nucleate hundreds of *motile cilia* per cell that beat vigorously in a whip-like motion to generate directional fluid flow across tissue surfaces. Thus, multiciliated cells (MCCs) are key players in respiration, neurogenesis and fertility. The motile cilium, composed of an axonemal 9+2 microtubular shaft with dynein arms and anchored by a basal body (BB) at the apical membrane, is a highly complex nanomachine with over 600 different proteins required for its assembly, structure and function.^19, 20^ Dysfunctional ciliogenesis underlie diverse human diseases, including hydrocephalus, hippocampal dysgenesis, PCD, Bardet–Biedl syndrome, asthma, anosmia, chronic obstructive pulmonary disease (COPD) and sterility.

MCCs have crucial roles in the mucosal defense of respiratory health, as they are essential for constantly cleaning the airways from inhaled pollutants, pathogens, allergens, dust and debris by a process called mucociliary clearance. Conducting airways are lined by polarized pseudostratified epithelium containing MCCs as well as secretory, goblet, neuroendocrine and adult stem cells.^21^ Impaired mucociliary transport caused by defective MCCs, either acquired or due to genetic ciliopathies like PCD, results in chronic airway infections, with airway remodeling and secondary alveolar destruction causing emphysema and COPD.^22^

**Figure 1:**
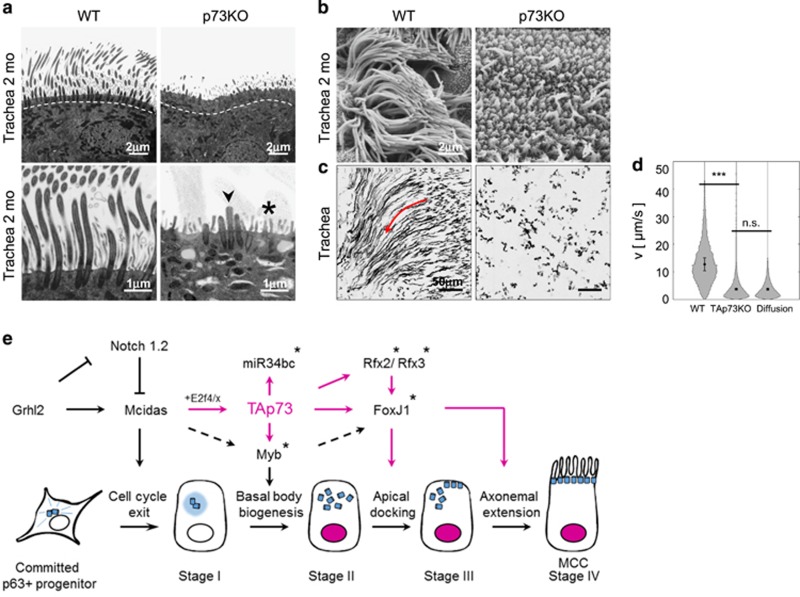
Selective absence of p73 in mice results in severe abnormalities of ciliogenesis in the airways. (**a**) Transmission electron microscopy and (**b**) scanning electron microscopy of the trachea of p73 knockout (p73KO) and control (WT) mice at 2 months of age. The p73-deficient mice show highly defective, rare, short, stump-like cilia (arrow). Asterix, microvilli. TAp73KO mice have a similar phenotype. (**c**) Measuring mucociliary transport. Aggregated fluorescent bead trajectories in control (WT) *versus* TAp73KO tracheae. WT trajectories (red arrow) follow a directional flow field that is completely lost in knockout tracheae. (**d**) Distribution of particle velocities (measured as *μ*m/s) from WT, TAp73KO and WT diffusion control (formalin-fixed) tracheae as in panel c. (**e**) Proposed role of TAp73 as master transcriptional regulator of motile multiciliogenesis in airways, directly controlling Fox, Rfx2/3, Myb and miR34, plus a plethora of ciliary structure/motility genes. Mcidas is upstream of TAp73. Modified with permission from Nemajerova *et al.*, 2016. Asterix indicates genes directly regulated by TAp73. n.s. nonsignificant

Focusing on airways, we found that all p73KO mice show severe respiratory distress already by 3 weeks of age, with constant coughing and sneezing due to chronic upper and lower respiratory tract infections including purulent sinusitis/rhinitis. Chronic bronchitis leads to bouts of pneumonia and over time causes inflammation-mediated secondary tissue destruction and lung emphysema. Isoform-specific TAp73KO mice also develop similar airway pathology and impaired pulmonary function. Wild-type (WT) epithelium strongly expresses p73 in the nucleus of suprabasal MCCs characterized by long abundant cilial ‘brooms’ at their apical cell surface. In contrast, p73KO and TAp73KO MCCs exhibit marked reduction in cilia number and length (i.e. drastically fewer and much shorter cilia) by light, electron and scanning microscopy (Figures 1a–d). The defect encompasses the entire upper and lower airways with the functional consequence of complete loss of mucociliary transport.^18^ This severe ciliary phenotype is also recapitulated in organotypic cultures, generated by isolating tracheal progenitors and triggering their mucociliary differentiation via air–liquid interface (ALI) conditions.^23, 24^ This also ensures that the ciliary phenotype seen *in vivo* is not simply due to secondary effects of inflammation. In sum, p73 is essential for the ciliation process in MCCs.

## TAp73 transcription is required for MCC BB docking and axonemal extension

Both mouse and analogous human tracheal epithelial cultures (TECs) induce TAp73 expression *early* after ciliogenesis initiation.^18^ When MCCs are quantified for sequential stages of ciliogenesis,^25^ the early stages I and II of p73KO MCCs, marked by centriolar amplification, appear relatively intact. In contrast, marked defects progressively worsen during stages III and IV. Final stage IV cells are strongly reduced, with reciprocal increase in unciliated and early-stage arrested cells. The largest reduction (by 90%) occurs in ‘fully ciliated’ cells, but even these have fewer and shorter cilia than WT MCCs. While the *number* of BBs (the complex cilial anchoring structures at the apical surface) is relatively intact, p73KO BBs show severe docking defects and fail to properly align at the apical membrane, and therefore are unable to extend axonemes. Overall, this indicates that p73 mainly functions after MCC fate specification and centriolar multiplication as the major upstream ciliogenesis regulator orchestrating BB docking, axonemal extension and motility.

**Figure 2:**
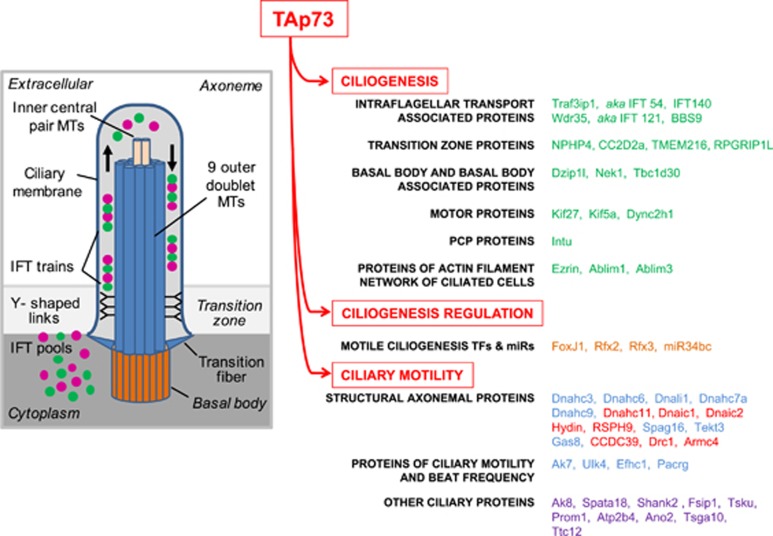
TAp73 is a crucial transcriptional regulator of multiciliogenesis. Scheme of cilium (left) and direct target genes of TAp73 in ciliogenesis (right). Genes were identified by RNA-seq analysis of WT *versis* TAp73KO airway cultures during MCC differentiation, combined with ChIP-seq analysis in TAp73*α*/*β-*expressing Saos-2 cells. In addition to central ciliogenic transcription factors and regulatory miR34bc, key structural and functional ciliary genes of every category including eight human PCD-associated genes indicated in red are among them. Modified with permission from Nemajerova *et al.*, 2016. IFT, intraflagellar transport; MTs, microtubules

Next, we compared RNA-seq expression profiles from WT and p73KO ALI cultures across differentiation with human TAp73 ChIP-seq data. This yielded a total of 1930 genes falling into three categories of (1) differentially expressed (DE) genes, (2) DE genes containing TP73-binding motifs and (3) bound DE genes containing a p73 motif in promoter/enhancer regions. Gene Ontology analysis showed significant enrichment of ciliogenesis genes, in fact, 12% of all 1236 DE genes at day 14 (full differentiation). The vast majority of these 155 ciliogenic genes are downregulated in p73KO, indicating a transactivating function for TP73. Fifty-two cilium DE genes are bound by TAp73 and contain the highly conserved TP73-binding motif (category 3). They include the key ciliogenic transcriptional regulators FoxJ1, Rfx2, Rfx3 and Myb,^19, 26^ and the regulatory microRNA miR34bc that enables BB docking.^27^ Moreover, nearly 50 key structural and functional ciliary genes associated with intraflagellar transport, transition fibers, BBs, planar cell polarity and motility (with many dynein motor genes) are also among them. Eight of these genes are known to cause hereditary human ciliopathies when mutated (Figure 2, genes highlighted in red).

Finally, we asked how TAp73 itself might be regulated during multiciliogenesis and identified Mcidas (aka Multicilin), using comparative genomic analysis of published data sets. Previous work identified Multicilin, a small coiled-coil transcriptional coregulator, to be required for the initial stages of MCC differentiation. It binds to E2F4/5 to promote transcription of key genes in centriole biogenesis.^28, 29^ In support, ectopic expression of Mcidas in WT ALI culture at day 0 rapidly induced TAp73 expression, and reporter assays confirmed the direct Mcidas/E2F4 responsiveness of the TAp73 promoter (Figure 1e).

## TAp73 is necessary and sufficient to drive the full multiciliogenesis program

Finally, to prove that TAp73 alone, rather than in cooperation with ΔNp73, is necessary and sufficient to fully direct ciliogenesis, we performed rescue experiments in global p73KO (missing both isoform classes) ALI cultures. Indeed, lentiviral TAp73*α*, expressed in MCC-fated cells, completely rescued cilia biogenesis. Also, to establish that TAp73 lies upstream of all currently known transcriptional activators, we performed epistasis analyses for FoxJ1, given its essential role in the multiciliogenesis hierarchy. Indeed, the severe defect of global p73KO ALI cultures were 100% rescued by lentiviral *FoxJ1* expression restricted to MCCs.^18^

In sum, our results place *TAp73* at the center of the gene-regulatory network controlling multiciliogenesis (Figure 2). Mechanistically, TAp73 directly activates key transcriptional and post-transcriptional regulators, most notably *FoxJ1*, *Rfx2*, *Rfx3*, *Myb* and miR34bc, as well as numerous structural and functional ciliogenesis genes. Our data further suggests that *Mcidas* regulates TAp73 in this process. This regulatory network is conserved between mouse and human.^18^

Two labs, focusing on ependymal biology, corroborated our findings. Using morphologic analysis of p73KO brains, they described cilial denudation of ependymal cells plus disorganization of the neurogenic niche cytoarchitecture (’pinwheels’), which lies directly underneath the ependymal layer.^15, 17^ Specifically, p73 deficiency impairs maturation, planar cell polarity and ciliogenesis of ependymal cells and thereby disturbs the neurogenic capacity of the subventricular zone.^15^

Moreover, a direct link between p73 and FoxJ1 was also independently identified by the Pietenpol lab.^16^ The authors found defects in p73-deficient multiciliated cells in the upper and lower respiratory tract, ependyma, oviduct and testis and used a ChIP-seq/RNA-seq approach on mouse tracheas to identify a broad p73-dependent ciliogenic program. And similar to our observations, ectopic TAp73*β* expression in mouse TEC cultures induced *FoxJ1* expression. Interestingly, using co-immunofluorescence these investigators also described a subset of p63-positive basal cells to coexpress p73, raising the possibility that p73 might also have some role in MCC fate specification and maintenance of the basal stem cell pool.^16^ More work is needed to resolve the meaning of this double-positive sub-population in tracheal epithelium. Now that TAp73 is the new master regulator of motile multiciliogenesis, future work will also need to clarify whether ΔNp73 has a role in ciliated epithelium, and if so, define its nature and whether or not it cooperates with TAp73 in cilial biology.

## The p53 family members in the control of cellular metabolism

The p53 family is well known for its tumor suppressor role that, to a different extent, is exerted by the full-length isoforms of all family members. Similar to p53KO mice,^30^ although only to a moderate degree, mice lacking TAp73 also develop spontaneous tumors^9^ and display increased susceptibility to chemical-induced carcinogenesis.^9, 31^ Lack of TAp63 isoforms has been associated with increased metastatic propensity of cancer cells, leading to the proposal of TAp63 as a tumor metastasis suppressor.^32, 33, 34^ All three family members, p53, TAp73 and TAp63, react to DNA damage, and upon severe or sustained genotoxicity promote cell death or senescence.^35^ These responses represent an effective barrier to eliminate damaged cells, thus limiting the inappropriate accumulation of cells that might initiate malignant development. In conditions of mild or short-term stress, p53 family members rather exert a repair response such as DNA repair or antioxidant defense to limit damage and restore physiological cell activity. Metabolic stress, nutrient or oxygen deprivation enable a p53 family-mediated adaptive response, in which metabolic adaptation and rebalancing of anabolism/catabolism coordinate a response to cope with such adverse conditions. While in physiologic conditions these functions preserve cellular and organ integrity, in cancer they might contribute to the development of the disease and to reduced therapeutic responses.

The dynamic, flexible and adaptable stress responses by p53 family members highlight the general complexity of the tumor suppressor mechanisms. However, they also suggest a rationale for the observed overlap between the contribution of these genes to the control of tumor development and to the maintenance of normal cellular homeostasis and organ development, such as multiciliogenesis. This is particularly remarkable for the p63 and p73 genes, which exert pivotal developmental functions. The contribution of TAp73 and the N-terminal truncated isoform of p63 (ΔNp63) to ciliogenesis and epidermal development, respectively, might indeed partially rely on their ability to control cellular metabolic homeostasis. Hence, conserved molecular mechanisms in different biological contexts lead to different outcomes. In this section, we will summarize the recent findings on p63 and p73 control of cellular metabolism, with a particular focus on glucose metabolism. We will also discuss the contribution of these processes to developmental and tumor-related functions of this gene family.

## TAp73 coordinates metabolic response with oxidative stress and amino-acid deprivation

**Figure 3:**
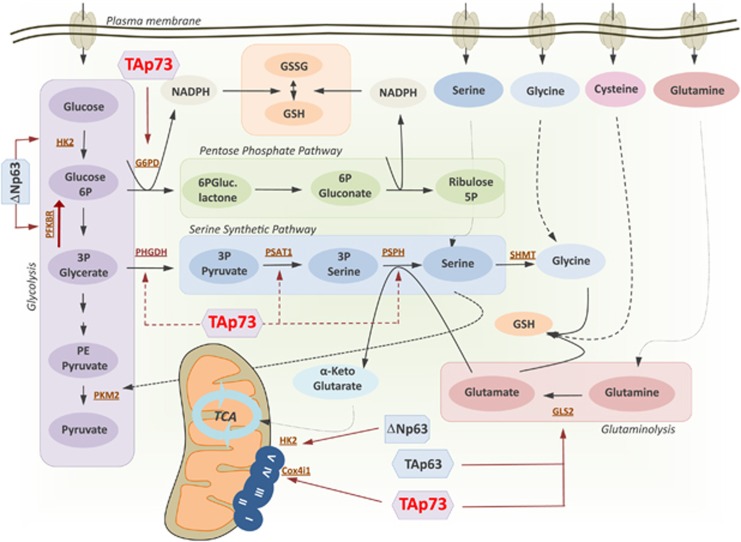
Influence of p73/p63 on the major metabolic pathways. P63 and p73 both promote a general increase in glucose catabolism, resulting in an increase in diverting anabolic pathways and mitochondrial respiration. Activation of serine biosynthesis and the PPP by TAp73, ΔNp63 and TAp63 facilitates the antioxidant response through increased synthesis of GSH and regeneration of reduced GSH. Conversely, a reduced function of TAp73 and ΔNp63 negatively influences mitochondrial respiration, leading to electron leaks and oxidative stress. Overall, p53 family member function on cellular metabolism appears a critical process for maintaining the cellular redox balance, which has important implications for a number of different biological processes in tumor suppression, stress response and development. GSSG, oxidized GSH

Glucose represents a major source of carbon and energy for mammalian cells. Thus, glycolysis is a critical metabolic pathway for virtually any cell type in physiological conditions and in disease. Cancer cells tend to rewire their metabolism, strongly increasing the rate of the glycolytic flux and converting the final product pyruvate to lactate rather than to acetyl-CoA. This shift produces what is defined as ‘aerobic glycolysis’ or ‘Warburg effect’ and is typically associated with a reduced tricarboxylic acid cycle (TCA) and thus much less efficient energy production. Despite the apparent poor ATP efficiency, this shift is important for cancer cells to refuel diverting anabolic pathways (Figure 3). Recent evidence suggests an expected role of TAp73 in promoting glycolytic flux.^36, 37^ High-throughput metabolomics in SaOs-2 human osteosarcoma cells unveiled increased rates of glycolysis, higher amino-acid uptake and increased levels and biosynthesis of acetyl-CoA following TAp73 overexpression. Remarkably, this metabolic shift correlated with an extensive TAp73-mediated upregulation of several anabolic pathways including for serine and glycine (SG),^38^ polyamine and the biosynthesis of membrane phospholipids.^36, 37^ Although apparently contradictory with the tumor suppressor role of TAp73, this metabolic effect does not really recapitulate the canonical cancer-associated metabolic changes, but rather suggests a role for TAp73 in promoting adaptive cellular mechanisms to stress conditions. In fact, the TAp73-dependent increase in glycolytic flux was also associated with an increase in both lactate and TCA cycle intermediates, indicating that TAp73 overexpression favors a general promotion of glucose metabolism towards production of energy and activation of anabolism.

One of the major aspects of TAp73's influence on cellular anabolism is its ability to promote SG biosynthesis.^38^ Serine represents a cellular metabolic hub, which provides precursors for the biosynthesis of amino acids, nucleic acids, lipids, ATP and antioxidative defense.^39^ Hyperactivation of SG biosynthesis and overexpression (amplification) of the respective metabolic enzymes are indeed observed in cancer.^40, 41^ TAp73 promotes synthesis of SG producing metabolic intermediates required for glutathione (GSH) production and therefore antioxidant defense. Interestingly, TAp73 deficiency sensitizes cells to SG deprivation, effectively creating a vulnerability in cancer cells. Mechanistically, TAp73 promotes expression of glutaminase-2, which converts glutamine to glutamate, which in turn promotes SG biosynthesis. Notably, both glutamate and glycine represent substrates for GSH synthesis. Thus, upon SG starvation TAp73 deficiency prevents a response for restoring proper antioxidant defense, resulting in proliferation arrest. Effectively, in these specific conditions the tumor suppressor TAp73 favors adaptation of cancer cells to adverse condition. A role for TAp73 in antioxidant response has also been reported by other studies.

TAp73 also regulates glucose metabolism to control the pentose phosphate pathway (PPP) and antioxidant capacity.^37, 42^ The PPP pathway is equally important for the antioxidant response, as NADPH produced in this pathway is required to regenerate reduced GSH.^43^ TAp73 transcriptionally promotes expression of the rate-limiting enzyme of PPP, glucose-6-phosphate dehydrogenase (G6PD). Remarkably, the proliferation arrest produced by TAp73 depletion can be restored by G6PD re-expression or by treatment with antioxidants such as *N*-acetylcysteine.^42^ This major regulatory role of TAp73 does not clash with its tumor suppressor ability, but rather represents a physiological protective mechanism exerted by TAp73. TAp73 deficiency indeed correlates with premature senescence and accelerated ageing in mice as a consequence of altered mitochondrial function and antioxidant balance.^44^ TAp73 transcriptionally controls the expression of mitochondrial complex IV subunit cytochrome *C* oxidase, subunit 4 (Cox4i1). Thus, TAp73 deficiency results in reduced cellular ATP levels, oxygen consumption and mitochondrial complex IV activity, with increased ROS production and oxidative stress sensitivity. Overall this leads to premature ageing of TAp73KO mice and accelerated senescence of TAp73KO MEFs. Importantly, this last phenotype can be reverted by a combination of antioxidants.^44^ This homeostatic role of TAp73 in regulating antioxidant defense preserves cells from premature senescence and might produce a vulnerability in cancer cells. This represents an instance in which the cancer cell hijacks a physiological protective function of a tumor suppressor gene. However, the role of TAp73 in regulating cancer metabolism has not been fully dissected yet and the scenario might be much more complex than what has been revealed so far, considering also the interplay of TAp73 with other pathways such as HIF-1 signaling.^31, 45^

TAp73 also exerts pivotal and exclusive roles in development. It is indeed remarkable that both p73KO and selective TAp73KO mice display severe morphological defects in the central nervous system, such as cortex hypoplasia and hippocampal dysgenesis.^2, 9^ Several mechanisms have been ascribed to p73's role in the brain; however, whether and how metabolic effects are responsible for this is still under investigation. Metabolomics analysis of TAp73KO and ΔNp73KO primary cortical neurons indicated that TA and ΔN isoforms have opposite effects on glycolytic pathways and mitochondrial respiration in neurons.^46^ However, the molecular basis for these effects and the contributions of p73-dependent metabolic changes to development and homeostasis such as neurodevelopment and multiciliogenesis will require further studies.

## Cellular metabolism is also controlled by p63 in epidermal development

Similarly to *TP73*, *TP63* gene gives rise to a wide number of isoforms, which include N-terminal alternative transcribed isoforms (TAp63 and ΔNp63) and C-terminal alternative spliced proteins (*α*, *β* and *γ*). A major aspect in development is the key function of the N terminally truncated isoform of p63 (ΔNp63) in stratified epithelial morphogenesis and homeostasis. Expression of ΔNp63 is critical for maintenance of stem cell-like properties and high proliferative status in basal keratinocytes. Control of cell cycle, cell adhesion and other mechanisms have been ascribed to this role.^47^ However, recent emerging evidence indicates a specific requirement for ΔNp63 for maintenance of an active metabolic status of proliferating keratinocytes (Figure 3). Depletion of ΔNp63 in human proliferating keratinocytes indeed leads to reduction of glycolytic rate and mitochondrial respiration.^48^ Altered mitochondrial function of ΔNp63-depleted keratinocytes is associated with mitochondrial hyperpolarization and increased intracellular ROS. Mechanistically, Viticchie *et al.*^48^ demonstrated that ΔNp63 physically binds to the hexokinase II (HK2) promoter and thus induces its transcriptional activation. The hexokinase family includes four isoforms that catalyze the first step of the glycolytic pathway converting glucose to glucose-6-phosphate. The preferential mitochondrial membrane localization of HK2 at voltage-dependent anion channels provides access to ATP generated by oxidative phosphorylation. Impairment of HK2 therefore might result in alteration of ADP/ATP turnover, impairment of the respiratory chain and electron leak. ΔNp63-depleted keratinocytes could restore their proliferation ability upon re-expression of HK2. Control of the keratinocyte glycolytic flux by ΔNp63 was confirmed in a second study, and this was associated with transcriptional regulation of 6-phosphofructo-2-kinase/fructose-2,6-biphosphatase 3, a dual enzyme with a 6-phosphofructo-2-kinase activity catalyzing synthesis of fructose-2,6-bisphosphate (F2,6BP) and a fructose-2,6-biphosphatase activity catalyzing the degradation of F2,6BP. PFKB3 is a major regulator of glycolysis; F2,6BP indeed acts as an allosteric activator of phosphofructokinase 1 (PFK1), the major rate-limiting enzyme of glycolysis. Hence, the *Δ*Np63/PFKB3 axis determines the rate of activation of glycolytic flux, thereby tuning the proliferation potential of basal keratinocytes. Aberrant expression of PFKB3 was indeed found in skin hyperproliferative conditions such as psoriatic epidermis, although the current data do not clarify whether accumulation of PFKB3 is causative of the pathological conditions or a simple consequence of the expansion of the proliferative compartment. Remarkably, these data all together indicate that in different cellular and biological contexts, the p53 family conserves this critical role of maintaining a healthy cellular oxidative balance. This function is also displayed by TAp63 whose activation promotes glycolytic flux and a slightly reduced TCA flux, thereby diverting glycolytic intermediates to anabolic pathways such as PPP and SG.^49^ The result of this metabolic control by TAp63 is again increased biosynthesis and regeneration of cellular GSH, which might have pivotal roles in different biological contexts.

## Other non-oncogenic functions of p73: fertility, neurodevelopment and inflammation

The complexity of p73 functions clearly emerges from the phenotypes of the KO mouse models, which show a range of defects that include infertility, an altered immune system and impaired neurological development. The control of genomic stability is central to all family members, and the genomic instability associated with TAp73 deficiency is not only important in tumorigenesis but also influences maturation of oocytes. In addition to cilia-deprived oviducts with severe transport defects, altered segregation of sister chromatids during meiosis leads to poor quality eggs and consequent female infertility. TAp73 controls the spindle assembly checkpoint (SAC) that prevents aberrant anaphase by inhibiting its onset until all chromosomes are properly attached to the spindle. SAC blocks progression of the cell cycle by inhibiting CDC20 and the anaphase-promoting complex. This guarantees a correct segregation of the sister chromatids. TAp73 physically binds to kinetochore proteins BUB1, BUB3 and BUBR1, allowing correct entry into anaphase.^9, 50^ Deregulation of this process in oocytes contributes to infertility: TAp73 deficiency in oocytes and cancer cells is indeed associated with aneuploidy.^51^ Similar to females but following a different mechanism, TAp73KO males also display reduced fertility. Seminiferous tubules of TAp73KO mice have indeed massive premature loss of immature male germ cells. The defect in sperm maturation is due to impaired cell–cell adhesion properties between developing germ cells and somatic Sertoli nurse cells. Mechanistically, TAp73 controls a transcriptional program in male germ progenitors that governs orderly spermatogenesis within the multilayered germinal epithelium.^52, 53^

Originally, the reduced fertility of the p73KO genotype was interpreted as defects in sensory and hormonal pathways contributing to behavioral phenotypes that preclude mating.^2^ However, this mechanism was later disproven, as TAp73KO mice displaying normal sensory and hormonal abilities still present with severe infertility.^9, 54^ However, alterations in sensory and hormonal pathways are part of the wide range of neurological defects displayed to different degrees by the Trp73KO mouse models. Anatomically, p73KO mice display severe hydrocephalus and hippocampal dysgenesis characterized by an unusual structural organization of the CA Summer sectors and the dentate gyrus (DG). TAp73KO mice display a less severe phenotype, but abnormal hippocampal development (truncation of the lower blade of the DG) still occurs. However, reduced cortical thickness and hydrocephalus is specific for p73KO mice, indicating that the concurrent loss of all TAp73 and ΔNp73 isoforms produce a much more severe phenotype than the addition of the individual phenotypes of the isoform-selective KO models. ΔNp73 mice indeed display only a marginal reduction of cortical thickness but no hydrocephalus.^55^

Different mechanisms underlying p73's contribution to neuronal development have been described. A general consensus exists on the role of TAp73 in the maintenance of embryonal and adult neural stem cells. TAp73KO neurospheres display defects in growth, associated with S-phase block and accelerated cellular senescence. Accordingly, p73-deficient mice (p73KO and TAp73KO) have significant depletion of stem cell reservoirs in the subgranular and subventricular zones.^11, 12, 13, 14, 56^ TAp73 deficiency also impairs neuronal differentiation. p73KO neuronal stem cells fail to fully differentiate and exhibit dendritic arborization and reduced synaptic connectivity. In this context, miR-34a appears to be a major transcriptional target of TAp73 for neuronal differentiation. *In vivo* evidence indicates a significant contribution of the TAp73/miR-34a axis to the neurological phenotype of p73 mouse models,^57^ and miR-34a KO mice likewise show significant reduction of precursors in the DG and subgranular zone.^58, 59^ However, the recent establishment of a role for TAp73 in governing ciliogenesis leads to a revision of the biological and molecular aspects underlying the fertility and neurological phenotypes of p73KO mice.

The influence of TAp73 on the immune response has been the focus of some studies, but how important its contribution is in the response to pathogens and cancer immunity remains to be determined. TAp73 has a role in macrophage polarization, as TAp73 deficiency prolongs maintenance of the M1 effector phenotype at the expense of the M2 phenotype. Altered M1/M2 macrophage polarization impairs the resolution of an inflammatory response. Indeed, lethal doses of lipopolysaccharide in TAp73KO mice leads to higher blood levels of proinflammatory cytokines and lethality.^60^ The original p73KO mouse showed recurrent infections, such as severe rhinitis and purulent otitis media. Massive neutrophil infiltrations are persistent from young age through adulthood in these anatomical sites, leading to chronic, bilateral rhinitis, otitis, periorbital edema and conjunctivitis. However, despite these signs of severe inflammation and infection, lymphoid or granulocyte populations appear normal in p73KO mice.^2^ Whether and how the altered macrophage polarization worsens the recurrent airway infections in p73KO and TAp73KO mice with their defective mucociliary clearance remains to be determined.

## Complex interplay between p73 and the other family members: structural insights

**Figure 4:**
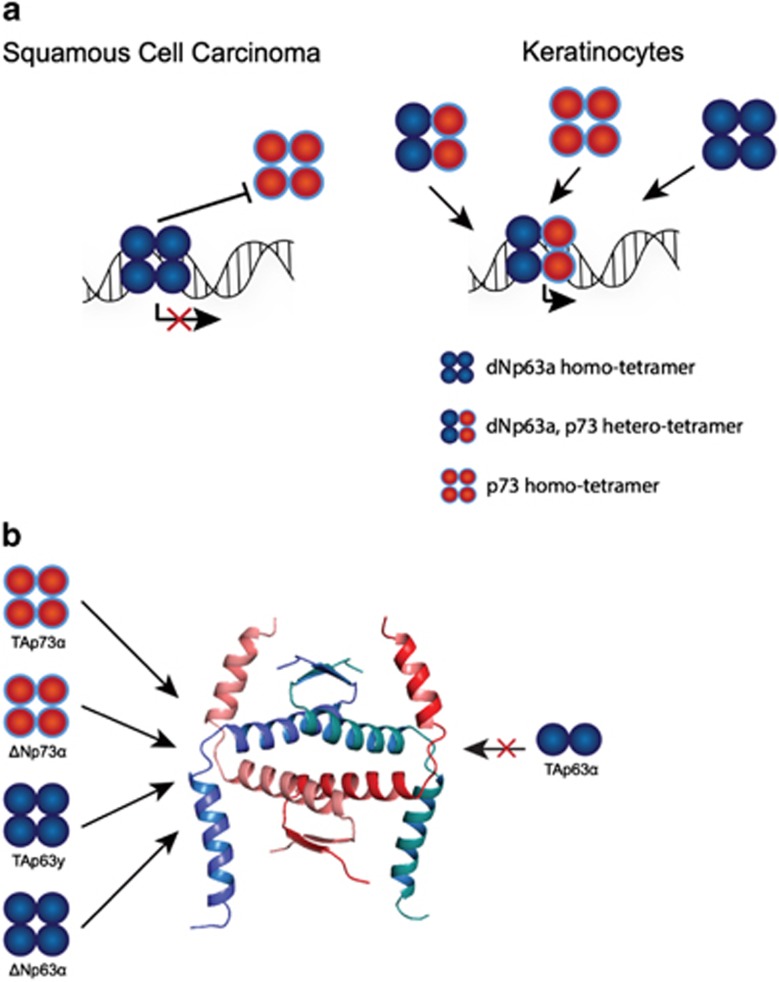
Tetramerization model of the p63/p73 interaction. (**a**) Heterotetramerization between p63 and p73 isoforms is possible in tissues where both proteins are coexpressed in the same cells at the same time. Squamous cell carcinomas express high levels of ΔNp63*α* as well as TAp73. The proapoptotic function of TAp73 seems to be repressed by the high abundance of ΔNp63 via promoter squelching, as knockdown of ΔNp63 leads to expression of Puma/Noxa with subsequent apoptosis. In basal keratinocytes ΔNp63*α* is also expressed at high levels. During differentiation a short burst of p73 expression can be observed. In addition, p63/p73 colocalization can be observed in some cells of the basal layer of the epidermis and the ORS of hair follicles of mouse skin. (**b**) Isoforms of p63 and p73 that are available for heterotetramer formation. P73 is a constitutive tetramer; therefore, all isoforms can participate in heterotetramer formation. In contrast, inactive TAp63*α* is a closed dimer with a buried tetramerization domain. All other p63 isoforms either lacking the N-terminal TA domain (ΔN isoforms) or the C-terminal inhibitory domain (TID, isoforms *β*, *γ*, *δ* and *ε*) are tetramers and therefore can participate in heterotetramer formation

All three members of the p53 family share a highly conserved DNA-binding domain that is the basis for a significant overlap in target genes and functional redundancies. Moreover, the complexity of biological functions in this family is further enhanced by the formation of oligomers. All family members can form tetramers through a highly conserved oligomerization domain. The architecture of this oligomerization domain is very similar between p63 and p73,^61, 62^ but differs from the oligomerization domain of p53. *In vitro* experiments with the isolated oligomerization domains have demonstrated that p63 and p73 form stable hetero-oligomers, while neither interacts with p53.^61, 62^ Surprisingly, a heterotetramer consisting of one p63 dimer and one p73 dimer is more stable than the two individual homotetramers. Structure determination of a heterotetramer of two p63 and two p73 molecules elucidated the basis for this interaction, revealing additional hydrophobic contacts that stabilize the heterotetramer^63^ (Figure 4). A potential specific function for the heterotetramer requires cells that coexpress both p63 and p73. Immunostainings of skin of 5-day-old mice (P5) has indeed shown that in the basal layer of the epidermis and in the outer root sheath (ORS) of hair follicles double-positive cells exist.^63^ While more cells in the basal layer and hair follicles show strong p63 expression, p73 expression seems to be confined to a smaller number of cells. In primary human keratinocytes, again strong p63 expression but only weak p73 expression can be detected, which however can be increased upon differentiation using high concentrations of calcium.^64^ Co-immunoprecipitation experiments revealed that in these coexpressing keratinocytes heterotetramers are formed.^63^ The existence of heterotetramers bound to DNA has also been suggested by a genome-wide mapping of p63 and p73 target sites in ME180 human cervical carcinoma cells.^65^ This study has revealed that the binding profile for p73 is indistinguishable from a previously determined binding profile of p63 in the same cell type.^66^ Sequential chromatin immunoprecipitation experiments further suggested that p63 and p73 co-occupy DNA target sites, arguing for the formation of heterotetramers *in vivo*.

Coexpression of p63 and p73 has also been reported in head and neck squamous cell carcinoma cells, a group of tumors derived from the basal epithelia of the aerodigestive mucosa.^67^ These cancer cells are characterized by high levels of the ΔNp63*α* protein.^68^ Interaction studies have shown that ΔNp63*α* interacts directly with transcriptionally active TAp73*β*, and that the transcriptional and tumor suppressor activity of this p73 isoform is inhibited by overexpression of *Δ*Np63*α*.^67^ In these cells, p63 knockdown leads to apoptosis through the expression of PUMA and NOXA. Further mechanistic studies have suggested, however, that the inhibitory effect is most likely caused by direct competitive binding of ΔNp63*α* to promotor sequences rather than by its binding to TAp73*β* protein.^63^

Interactions between p63 and p73 can in principle occur with all isoforms, with the exception of TAp63*α*. This isoform is very highly expressed in primary oocytes that are arrested in meiosis I^54^ in the ovary, as well as in fibronectin-positive dermal sheath cells of the hair follicle, in a sub-population of NCAM-positive dermal papillae cells^69^ and in hepatocytes where its overexpression induces liver steatosis through activation of IKK*β*.^70^ In healthy oocytes TAp63*α* forms closed, transcriptionally inhibited dimers^71^ through the formation of a *β*-sheet between the N-terminal transactivation domain and the C-terminal transactivation inhibitory domain with the central oligomerization domain.^72^ In contrast to TAp63*α*, TAp73*α* forms an open tetramer despite high sequence identity with TAp63*α* including the N- and C-terminal domains.^73^ Upon DNA damage, the activity of TAp63*α* is regulated by kinases that phosphorylate^74^ and open the closed inhibited dimeric state, thereby allowing the formation of active tetramers that eliminate the damaged oocyte.^72^ For p73, different transcriptional activities of individual C-terminal splice variants have been reported.^3^ However, these different C termini do not seem to regulate their activity by modulating their oligomeric state.^73^ Instead, differential recruitment of cofactors and other proteins that modulate their transcriptional activity at specific promoter sites, as well as differential intracellular stability might be mechanisms determining how p73's activity is regulated, derived from a combination of concentrations of its different splice forms and post-translational modifications.^5, 75, 76^

## Concluding remarks

The importance of the p53 family for tumor suppression has been thoroughly established during the past few decades, but the rapid development of several mouse models has also highlighted pivotal non-oncogenic roles for all family members, in particular for TAp63*α*, ΔNp63 and TAp73. The recent discovery of TAp73 as the master regulator of multiciliogenesis has opened alternative explanations for some long-standing perplexing questions in the field, as a molecular and biological foundation for the immunological, neurological and reproductive phenotypes displayed by p73-deficient mouse models. Together with the identification of critical roles in regulating cellular metabolism, this discovery has also layed open a substantial number of novel biological problems that might have important implications for human health. It will be critical to now investigate whether the p73-dependent defects in airway ciliogenesis are associated with human pathological conditions such as COPD or lung cancer. Indeed, the distinction between tumor-suppressive and non-cancer roles of TAp73 might not be as sharp and a wide overlap of similar functions in different contexts could be responsible for distinct phenotypes. This is particularly evident in the regulation of cellular metabolism, where for example the homeostatic control of physiological oxidative balance might have a role during cancer transformation or even, paradoxically, favor cancer progression in later stages of the disease. Another important aspect that recently emerged is the intrafamily interplay, with the ability of p63 and p73 to form genome-wide heterotetramers bound to DNA. The function of these two members might indeed need to be investigated globally, as the study of the two genes individually could provide only a restricted view of their complex functions.
